# The impact of genome‐wide association studies on the pathophysiology and therapy of cardiovascular disease

**DOI:** 10.15252/emmm.201506174

**Published:** 2016-05-04

**Authors:** Thorsten Kessler, Baiba Vilne, Heribert Schunkert

**Affiliations:** ^1^ Deutsches Herzzentrum MünchenKlinik für Herz‐ und KreislauferkrankungenTechnische Universität MünchenMunichGermany; ^2^ DZHK (German Center for Cardiovascular Research) e.V., partner site Munich Heart AllianceMunichGermany

**Keywords:** atherosclerosis, coronary artery disease, genome‐wide association studies, myocardial infarction, Cardiovascular System, Chromatin, Epigenetics, Genomics & Functional Genomics

## Abstract

Cardiovascular diseases are leading causes for death worldwide. Genetic disposition jointly with traditional risk factors precipitates their manifestation. Whereas the implications of a positive family history for individual risk have been known for a long time, only in the past few years have genome‐wide association studies (GWAS) shed light on the underlying genetic variations. Here, we review these studies designed to increase our understanding of the pathophysiology of cardiovascular diseases, particularly coronary artery disease and myocardial infarction. We focus on the newly established pathways to exemplify the translation from the identification of risk‐related genetic variants to new preventive and therapeutic strategies for cardiovascular disease.

**Glossary**
Coronary artery disease: Coronary artery disease is the manifestation of atherosclerosis in the coronary arteries, that is the vessels supplying the myocardium with oxygen and nutrients. Major complications of coronary artery disease include chest pain, myocardial infarction, arrhythmias and heart failure. It is one of the leading causes of death.
Extracellular matrix: The extracellular matrix is a conglomerate of molecules and cells of often different types. The fibroblasts are the main cell type involved in the formation of the extracellular matrix. In addition to the effects on tissue/organ physical properties, for example elasticity and stiffness, the extracellular matrix also has effects on the behaviour of imbedded cells, that is migration but also gene expression and differentiation.
Linkage disequilibrium: Linkage disequilibrium describes the occurrence that two alleles at different loci are not independently distributed in a population.
Precision medicine: The response to a treatment modality may vary, even in patients with the same diagnosis. Precision medicine aims to tailor healthcare to individual patients. It can involve molecular diagnostics but also laboratory tests, imaging, environmental analyses and large‐scale data analysis, for example pattern recognition.
Single Nucleotide Polymorphisms: Single nucleotide polymorphisms (SNPs) are variations in single nucleotides that occur in the genome with distinct frequencies. Association studies exploit the analysis of SNPs to investigate the association of these genetic variants with a particular disease.
Statins: Statins are drugs used to lower lipid levels, especially low‐density lipid (LDL) cholesterol. The molecular target of statins is the enzyme HMG‐CoA reductase, a key player in the endogenous production of cholesterol. Statins have proven efficacy in the reduction in both LDL cholesterol and cardiovascular events.
Systems medicine: Systems medicine is a novel discipline in biomedical research, closely related to systems biology. Systems medicine aims to study the individual in an integrated fashion, incorporating, for example, “‐omics” data, and biochemical and environmental interactions. As a further dimension, changes over time are included in the analyses. A major focus in systems medicine research is the development of computational models.

## Introduction

Atherosclerosis is a systemic disorder with high prevalence in both industrialized and developing countries. Its manifestations in the form of cerebrovascular or coronary artery disease (CAD) account for 46% of all deaths in Europe (Nichols *et al*, 2014). Although progress in cardiovascular medicine has substantially reduced the death toll in the past decades, deeper insight into the pathophysiology of atherosclerosis is mandatory to further improve strategies for the prevention and treatment. Epidemiological studies, especially the Framingham Heart Study, spearheaded the efforts to track down the causes for myocardial infarction (MI) and led to the identification of several predictors for CAD, for example hypertension and hypercholesterolaemia (Kannel *et al*, 1961) which, together with obesity, smoking and diabetes, are condensed under the term “modifiable risk factors”, as they can be addressed by lifestyle changes and therapeutic interventions (Yusuf *et al*, 2004).

It is also known that CAD has a high heritability that is by far larger than the genetic influence on the modifiable risk factors (Marenberg *et al*, 1994). However, for many decades, the attempts to identify the genetic variants underlying this heritability were unsuccessful (Mayer *et al*, 2007). Over the last few years, methodological progress and collaborative efforts have changed this scenario. The invention of arrays for the low‐cost genotyping of hundreds of thousands of patient genomic markers, annotation of these markers in the human genome together with meta‐analyses of multiple case–control samples have enabled the identification of numerous genetic variants that are highly and reproducibly associated with CAD and MI risk.

Analysis of genotype frequencies with the aim to detect variants that occur at different frequency in cases and controls is now being carried out at the genome‐wide level. Variants found in such genome‐wide association studies (GWAS) to be associated with disease risk are not necessarily located in the coding regions. The fact that chromosomal loci tagged by respective variants do play a causal role in modulating risk is based on very strong statistical significance; however, the mechanisms involved remain often unclear. The hypothesis‐free method of the GWAS approach thereby differs from other emerging techniques such as exome sequencing (Table 1), where specific genes are being tested for association with disease. Moreover, the variant with the strongest association in a GWAS does not necessarily represent the causal one; it may rather be in linkage disequilibrium with a functionally relevant allele that is located in close proximity at this locus. Nevertheless, the GWAS approach has proven to be a powerful tool: as discussed in this review, more than 50 single nucleotide polymorphisms (SNPs) tagging distinct chromosomal regions have been annotated so far to be associated with CAD with high statistical significance. Here, we focus on variants that allow inference to the causal genes and molecular pathways and thus improve our understanding of the pathophysiology of CAD and MI, which may ultimately result in better treatment and prevention of these deadly diseases.

**Table 1:** Association approaches in cardiovascular research (*) can be grouped into haplotypes, which may allow to derive more genetic information or imputation of other SNPs; ^#^, can be further categorized if functional implications are known, for example effect of expression level = eSNP; ^+^; may vary in different tissues or conditions

	GWAS	EWAS	Exome array	Exome sequencing	Genome sequencing
Focus	Common SNPs*^#^	CpG methylation sites	Exonic variants	Coding sequences of all genes (high coverage)	Sequences of all genes (high coverage)
N of signals	~8·10^6^	~500·10^3^	~220·10^3^	~30·10^6^	3·10^9^
Coverage	Whole genome	Whole genome^+^	All genes	All genes	Whole genome
Costs per person [$]	~100	~300	~100	~600	> 1,000

bp, base pairs; EWAS, epigenome‐wide association study; GWAS, genome‐wide association study; N, number.

## Genome‐wide association studies in coronary artery disease and myocardial infarction

In 2007, the first GWAS were published for CAD, all of which identified a locus on chromosome 9p21 to be genome‐wide significantly associated with the disease (Helgadottir *et al*, 2007; McPherson *et al*, 2007; Samani *et al*, 2007). This locus became the first claim of what has later been called a gold rush of CAD genetics. Interestingly, the chromosome 9p21 locus still has an outstanding position as it depicts the genetic variant with the highest population‐attributable risk (Table 2; Schunkert *et al*, 2008).

**Table 2:** Loci identified to be associated with CAD/MI either by genome‐/exome‐wide association studies

Chr.	Lead SNP	AF	OR	Gene at chr. locus	Bioinf. annot.	HTN	LIP	References
1	rs11206510	T (0.82)	1.08	*PCSK9*	No data		+	Myocardial Infarction Genetics Consortium (2009), Abifadel *et al* (2003), Cohen *et al* (2006), Teslovich *et al* (2010)
	rs17114036	A (0.91)	1.17	*PPAP2B*	No data			Schunkert *et al* (2011)
	rs17465637	C (0.74)	1.14	*MIA3*	*MIA3*,* AIDA*,* C1orf58*			Samani *et al* (2007), Schunkert *et al* (2011)
	rs599839	A (0.78)	1.11	*SORT1*	*SORT1*		+	Schunkert *et al* (2011), Teslovich *et al* (2010), Samani *et al* (2007)
	rs4845625	T (0.47)	1.06	*IL6R*	*IL6R*,* ATP8B2*,* CHTOP*,* UBAP2L*			CARDIoGRAMplusC4D Consortium (2013)
2	rs6544713	T (0.30)	1.06	*ABCG5/ABCG8*	No data		+	Schunkert *et al* (2011), Teslovich *et al* (2010), IBC 50K CAD Consortium (2011)
	rs6725887	C (0.15)	1.14	*WDR12*	NA			Schunkert *et al* (2011), Myocardial Infarction Genetics Consortium (2009)
	rs515135	G (0.83)	1.07	*APOB*	No data		+	CARDIoGRAMplusC4D Consortium (2013), Teslovich *et al* (2010)
	rs2252641	G (0.46)	1.06	*ZEB2*	No data			CARDIoGRAMplusC4D Consortium (2013)
	rs1561198	A (0.45)	1.06	*VAMP5‐VAMP8‐GGCX*	*VAMP5/8*			CARDIoGRAMplusC4D Consortium (2013)
3	rs2306374	C (0.18)	1.12	*MRAS*	*MRAS*,* CEP70*			Erdmann *et al* (2009), Schunkert *et al* (2011)
4	rs7692387	G (0.81)	1.08	*GUCY1A3*	no data	+		CARDIoGRAMplusC4D Consortium (2013), Erdmann *et al* (2013), International Consortium for Blood Pressure Genome‐Wide Association Studies (2011)
	rs1878406	T (0.15)	1.10	*EDNRA*	NA			CARDIoGRAMplusC4D Consortium (2013)
	rs17087335	T (0.21)	1.06	*REST‐NOA1*	NA			Nikpay *et al* (2015)
5	rs2706399	G (0.51)	1.07	*IL5*	NA			IBC 50K CAD Consortium (2011)
	rs273909	C (0.14)	1.07	*SLC22A4‐A5*	No data			CARDIoGRAMplusC4D Consortium (2013)
6	rs12526453	C (0.67)	1.10	*PHACTR1*	No data			Schunkert *et al* (2011), Myocardial Infarction Genetics Consortium (2009)
	rs17609940	G (0.75)	1.07	*ANKS1A*	NA			Schunkert *et al* (2011)
	rs12190287	C (0.62)	1.08	*TCF21*	No data			Schunkert *et al* (2011)
	rs3798220	C (0.02)	1.51	*LPA, SLC22A3, LPAL2*	*LPA*		+	Tregouet *et al* (2009), Schunkert *et al* (2011), Teslovich *et al* (2010)
	rs10947789	T (0.76)	1.07	*KCNK5*	No data			CARDIoGRAMplusC4D Consortium (2013)
	rs4252120	T (0.73)	1.07	*PLG*	*PLG*,* LPAL2*			CARDIoGRAMplusC4D Consortium (2013)
7	rs10953541	C (0.80)	1.08	*BCAP29*	No data			Coronary Artery Disease C4D Genetics Consortium (2011)
	rs11556924	C (0.62)	1.09	*ZC3HC1*	*ZC3HC1*			Schunkert *et al* (2011)
	rs2023938	G (0.10)	1.08	*HDAC9*	No data			CARDIoGRAMplusC4D Consortium (2013)
	rs3918226	T (0.06)	1.14	*NOS3*	*NOS3*			Nikpay *et al* (2015)
8	rs2954029	A (0.55)	1.06	*TRIB1*	No data		+	IBC 50K CAD Consortium (2011), CARDIoGRAMplusC4D Consortium (2013), Teslovich *et al* (2010)
	rs264	G (0.86)	1.11	*LPL*	*LPL*		+	CARDIoGRAMplusC4D Consortium (2013), Teslovich *et al* (2010), Stitziel *et al* (2016)
9	rs4977574	G (0.46)	1.29	*9p21.3*	*ANRIL*			Samani *et al* (2007), McPherson *et al* (2007), Helgadottir *et al* (2007), Schunkert *et al* (2011), Holdt *et al* (2010)
	rs579459	C (0.21)	1.10	*ABO*	NA		+	Schunkert *et al* (2011), Teslovich *et al* (2010), Reilly *et al* (2011)
	rs111245230	C (0.04)	1.14	*SVEP1*	NA			Stitziel *et al* (2016)
10	rs2505083	C (0.38)	1.07	*KIAA1462*	*KIAA1462*			Erdmann *et al* (2009), Coronary Artery Disease C4D Genetics Consortium (2011)
	rs1746048	C (0.87)	1.09	*CXCL12*	NA			Samani *et al* (2007), Schunkert *et al* (2011)
	rs1412444	T (0.42)	1.09	*LIPA*	NA			Coronary Artery Disease C4D Genetics Consortium (2011)
	rs12413409	G (0.89)	1.12	*CYP17A1, NT5C2*	NA	+		Schunkert *et al* (2011), Newton‐Cheh *et al* (2009), Levy *et al* (2009)
11	rs974819	T (0.32)	1.07	*PDGFD*	No data			Coronary Artery Disease C4D Genetics Consortium (2011)
	rs964184	G (0.13)	1.13	*APOA1‐C3‐A4‐A5*	No data		+	Schunkert *et al* (2011), The TG and HDL Working Group of the Exome Sequencing Project, National Heart, Lung, and Blood Institute (2014), Do *et al* (2015, 2013)
12	rs10840293	A (0.55)	1.06	*SWAP70*	NA			Nikpay *et al* (2015)
	rs3184504	T (0.44)	1.07	*SH2B3, HNF1A*	*SH2B3*,* FLJ21127*,* ATXN2*	+	+	Schunkert *et al* (2011), Teslovich *et al* (2010), Newton‐Cheh *et al* (2009), Levy *et al* (2009), Gudbjartsson *et al* (2009)
	rs11830157	G (0.36)	1.12	*KSR2*	NA			Nikpay *et al* (2015)
13	rs4773144	G (0.44)	1.07	*COL4A1, COL4A2*	No data			Schunkert *et al* (2011)
	rs9319428	A (0.32)	1.06	*FLT1*	No data			CARDIoGRAMplusC4D Consortium (2013)
14	rs2895811	C (0.43)	1.07	*HHIPL1*	*YY1*			Schunkert *et al* (2011)
15	rs3825807	A (0.57)	1.08	*ADAMTS7*	*ADAMTS7*			Schunkert *et al* (2011), Reilly *et al* (2011), Coronary Artery Disease C4D Genetics Consortium (2011)
	rs17514846	A (0.44)	1.07	*FURIN‐FES*	*FURIN*,* MAN2A2*	+		CARDIoGRAMplusC4D Consortium (2013), International Consortium for Blood Pressure Genome‐Wide Association Studies (2011)
	rs56062135	C (0.79)	1.07	*SMAD3*	NA			Nikpay *et al* (2015)
	rs8042271	G (0.9)	1.10	*MFGE8‐ABHD2*	NA			Nikpay *et al* (2015)
17	rs216172	C (0.37)	1.07	*SMG6‐SRR*	NA			Schunkert *et al* (2011)
	rs12936587	G (0.56)	1.07	*PEMT, RASD1, SMCR3*	*TOM1L2*			Schunkert *et al* (2011)
	rs46522	T (0.53)	1.06	*UBE2Z, GIP, ATP5G1*	NA			Schunkert *et al* (2011)
	rs7212798	C (0.15)	1.08	*BCAS3*	NA			Nikpay *et al* (2015)
18	rs663129	A (0.26)	1.06	*PMAIP1‐MC4R*	NA			Nikpay *et al* (2015)
19	rs116843064	G (0.98)	1.14	*ANGTPL4*	NA		+	Teslovich *et al* (2010), Stitziel *et al* (2016)
	rs1122608	G (0.77)	1.14	*LDLR*	*SMARCA4*		+	Myocardial Infarction Genetics Consortium (2009), Schunkert *et al* (2011), Teslovich *et al* (2010), Do *et al* (2015)
	rs2075650	G (0.14)	1.14	*APOE*	*TOMM40*		+	IBC 50K CAD Consortium (2011), Teslovich *et al* (2010)
	rs12976411	A (0.91)	1.33	*ZNF507‐ LOC400684*	NA			Nikpay *et al* (2015)
21	rs9982601	T (0.15)	1.18	*MRPS6, SLC5A3, KCNE2*	No data			Myocardial Infarction Genetics Consortium (2009)
22	rs180803	G (0.97)	1.20	*POM121L9P‐ADORA2A*	NA			Nikpay *et al* (2015)

Chr., chromosome/chromosomal; SNP, single nucleotide polymorphism; AF, risk allele and its frequency; OR, odds ratio; HTN, associated with blood pressure; LIP, associated with LDL cholesterol/lipoprotein (a)/triglycerides; Bioinf. annot., bioinformatics annotation according to Braenne *et al* (2015) or others (Musunuru *et al*, 2010; Salvi *et al*, 2013); no data, no eQTL data or non‐coding variant; NA, not analysed.

**Figure 1:**
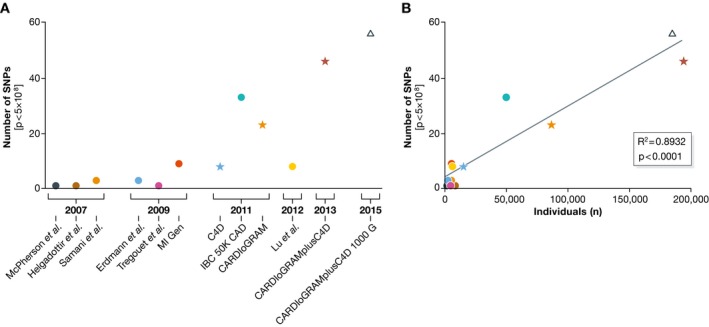
Numbers of individuals and SNPs investigated by GWAS influence the power for the detection of associated loci The number of investigated individuals in the discovery phases of the relative GWAS/meta‐analyses was plotted against the number of variants reaching genome‐wide level of significance in the overall analysis of the studies. Association between individuals in the discovery phase and the number of hits was evaluated by linear regression. *P* < 0.05 was considered as statistically significant. GraphPad Prism version 6.0c for Mac OS X (GraphPad Software, La Jolla, CA, USA) was used. (A) The number of SNPs detected at genome‐wide significant level for coronary artery disease in consecutive studies. (B) The number of SNPs detected with genome‐wide significance after replication correlates with the number of individuals included in the discovery studies. Symbols denote the numbers of genotyped SNPs [dots: ≤ 500,000 SNPs (Samani *et al*, 2007; McPherson *et al*, 2007; Helgadottir *et al*, 2007; Myocardial Infarction Genetics Consortium, 2009; Erdmann *et al*, 2009; Tregouet *et al*, 2009; IBC 50K CAD Consortium, 2011; Lu *et al*, 2012); asterisks: 2,500,000 SNPs (Coronary Artery Disease C4D Genetics Consortium, 2011; Schunkert *et al*, 2011; CARDIoGRAMplusC4D Consortium *et al*, 2013); arrow: 940,000 SNPs (Nikpay *et al*, 2015)].

In the first years of GWAS discoveries, identification of further loci came from individual genome‐wide association studies (Erdmann *et al*, 2009; Myocardial Infarction Genetics Consortium *et al*, 2009; Tregouet *et al*, 2009; IBC 50K CAD Consortium, 2011; Wang *et al*, 2011), whereas more recently the formation of large, international consortia has accumulated a sufficient statistical power for new discoveries. The joint forces of the CARDIoGRAM (Schunkert *et al*, 2011), C4D (Coronary Artery Disease C4D Genetics Consortium, 2011) and finally the CARDIoGRAMplusC4D (CARDIoGRAMplusC4D Consortium *et al*, 2013; Nikpay *et al*, 2015) consortium allowed the analysis of up to 180,000 individuals, about half of which had CAD. Interestingly, consequent studies revealed a strong relationship between the numbers of individuals investigated and the numbers of genome‐wide significant variants detected by the GWAS approach (Fig 1). This close correlation makes it likely that many more variants may eventually achieve genome‐wide significant association once even larger sample sets will be studied.

Another means to increase the power of GWAS is by denser granularity of SNPs. This progress was initially driven by better arrays allowing the genotyping of more and more SNPs. More recently, improvement came from imputation of SNPs based on the 1,000 genomes sequencing data set (1000 Genomes Project Consortium *et al*, 2012). It is now possible to study more than 15 Mio. distinct SNPs in human DNA for association with disease. Denser genotyping and higher numbers of study subjects increased the number of loci with genome‐wide significant association for CAD to 56 (CARDIoGRAMplusC4D Nikpay *et al*, 2015). Table 2 gives an overview about the loci thus far identified to be genome‐wide significantly associated with CAD by GWAS.

Almost all risk variants identified by GWAS are commonly found in European populations. This led to the unexpected finding that an average European individual carries tens of these risk alleles. Moreover, almost all are located in non‐coding parts of the genome, which suggests that these variants are more likely to affect gene regulation rather than protein structure. Indeed, annotation of the risk alleles revealed that the majority are located in chromosomal regions relevant for gene regulation. The identification of the underlying pathophysiological mechanism(s) thus requires annotation of the affected gene(s), which may be challenging (Braenne *et al*, 2015). The chromosome 9p21 locus is a prominent example: whereas initial studies focused on the two genes situated at the locus, *CDKN2A* and *CDKN2B,* current studies rather point to radically new disease mechanisms. Indeed, it appears that the risk/non‐risk alleles at the 9p21 locus relate to different isoforms of *ANRIL* and subsequently to preferred synthesis of non‐circular/circular forms of this long non‐coding RNA, which affect via ribosomal function the expression of multiple genes leading ultimately to opposing effects on cell proliferation and apoptosis (Holdt *et al*, 2010, 2013).

## Exome‐wide association study in coronary artery disease

The annotation to a gene is easier to achieve if the SNP leading a GWAS signal causes a mutation in the coding region presumably influencing protein function (Table 3). Important examples that will be discussed in more detail below include the *PCSK9* and *GUCY1A3* genes. Therefore, the idea arose to systematically investigate coding variants.

**Table 3:** Genes associated with CAD/MI with lead SNPs, or proxy SNPs of the respective lead SNP, causing a deleterious variation, and genes identified by beneficial/deleterious mutations to be associated with CAD/MI

Chr.	Gene	AA variation/type of mutation	Risk	References
1	*CCDC181*	p.F238I	↑	Braenne *et al* (2015)
	*LMOD1*	p.T295M	↑	Braenne *et al* (2015)
	*PCSK9*	p.S127R/p.F216L p.Y142X/p.C679X	↑ ↓	Abifadel *et al* (2003) Cohen *et al* (2006)
2	*WDR12*	p.I75V	↑	Braenne *et al* (2015)
	*TNS1*	p.W1197R	↑	Braenne *et al* (2015)
3	*MAP4*	p.V628L/p.S427Y	↑	Braenne *et al* (2015)
4	*GUCY1A3*	Nonsense/p.Gly537Arg	↑	Erdmann *et al* (2013)
7	*ZC3HC1*	p.R363H	↑	Braenne *et al* (2015)
	*NPC1L1*	Nonsense/frameshift/splice site	↓	Myocardial Infarction Genetics Consortium Investigators *et al* (2014)
8	*LPL*	p.D36N nonsense	↑ ↓	Stitziel *et al* (2016)
9	*SVEP1*	p.D2702G	↑	Stitziel *et al* (2016)
10	*BMPR1A*	p.P2T	↑	Braenne *et al* (2015)
11	*APOA5*	Non‐synonymous	↑	Do *et al* (2015)
	*APOC3*	Non‐synonymous/splice site/null	↓	The TG and HDL Working Group of the Exome Sequencing Project, National Heart, Lung, and Blood Institute (2014)
12	*HNF1A*	p.I27L	↑	Braenne *et al* (2015)
17	*LRRC48*	p.R191W	↑	Braenne *et al* (2015)
19	*LDLR*	Non‐synonymous/null	↑	Do *et al* (2015)
	*ANGPTL4*	p.E40K	↓	Stitziel *et al* (2016)
20	*MYH7B*	p.A25T	↑	Braenne *et al* (2015)
	*PROCR*	p.S219G	↑	Braenne *et al* (2015)

Chr., chromosome; AA, amino acid; ↑, variant increases risk; ↓, variant decreases risk.

See also Table 1 and Fig 3.

The results of the first exome‐wide association study on CAD have been just published (Stitziel *et al*, 2016). The authors included more than 72,000 cases and more than 120,000 controls, respectively. They confirmed signals in the *LPA* and *PCSK9* genes but also identified novel variants in genes thus far not associated with CAD: a missense variant in the *ANGPTL4* gene and a missense variant in the sushi, von Willebrand factor type A, EGF and pentraxin domain containing 1 gene (*SVEP1*). *SVEP1* was not associated with lipid levels, but with both systolic and diastolic blood pressure leading to an increase of 0.94 and 0.57 mmHg, respectively, in those carrying the risk allele (Stitziel *et al*, 2016). Moreover, SVEP1 has been investigated in septic shock and endotoxaemia (Nakada *et al*, 2015). Specifically, the inhibition of *SVEP1* expression by RNAi was found to regulate the expression of adhesion molecules on the surface of endothelial cells (Schwanzer‐Pfeiffer *et al*, 2010). One paper also identified SVEP1 as the physiological ligand of integrin α9β1. Integrins are key molecules in the interaction of different cell types through the extracellular matrix (ECM) such that it may be feasible that SVEP1 influences cell adhesion mediated by integrin α9β1 (Sato‐Nishiuchi *et al*, 2012), which could be of pathophysiological relevance in CAD.

## Pathophysiological insights

**Figure 2:**
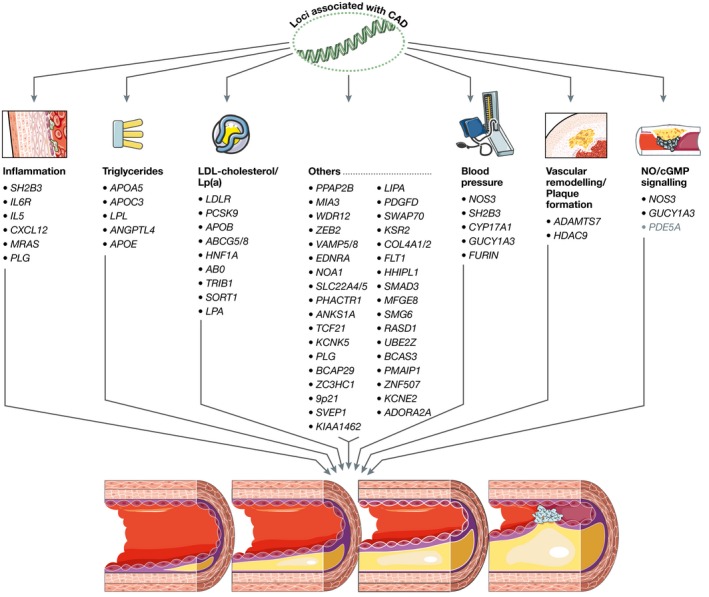
Genetic variation and pathophysiological pathways in atherosclerosis

**Figure 3:**
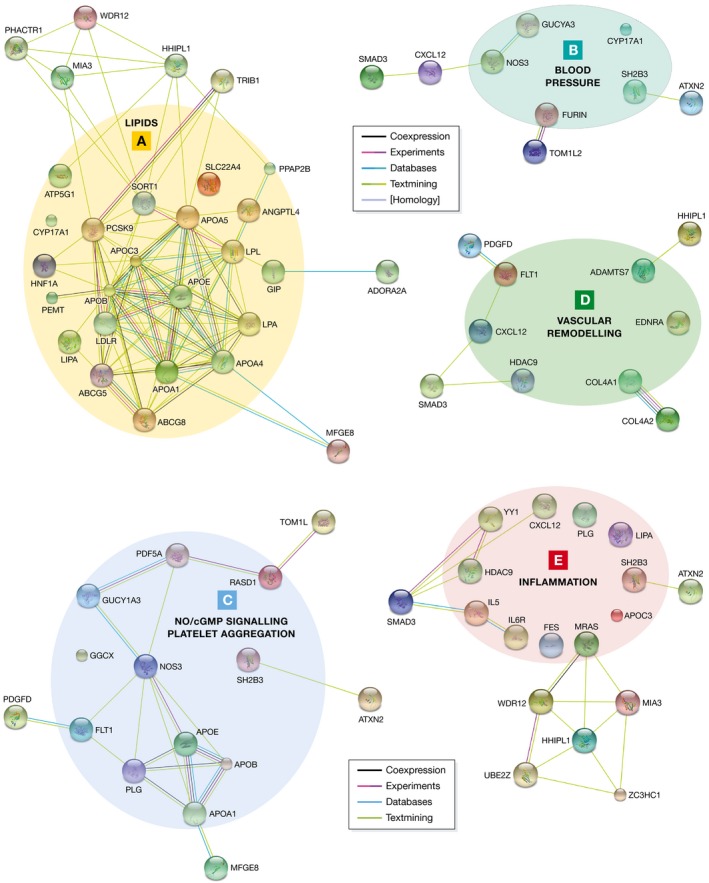
Genes involved in different pathophysiological pathways extracted from the 56 loci listed in Table 2 Functional annotations were collected from (i) the ConsensusPathDB database (http://consensuspathdb.org; Kamburov *et al*, 2013), (ii) the AmiGO 2 Gene Ontology (GO) browser (http://amigo.geneontology.org/amigo; Carbon *et al*, 2009), as well from (iii) the biomedical literature. Known and predicted associations among the genes within each functional category/pathway were retrieved from the STRING database (http://string-db.org; Franceschini *et al*, 2013) using default parameters. (A) Lipid metabolism. (B) Blood pressure. (C) NO‐cGMP signalling/platelet aggregation. (D) Vascular remodelling. (E) Inflammation.

Bioinformatics analyses and experimental studies revealed the involvement of the genes affected by risk alleles in different pathophysiological pathways (Figs 2 and 3). In the following paragraphs, we highlight the effects on LDL cholesterol and triglyceride metabolism, blood pressure, NO/cGMP signalling, vascular remodelling and inflammation.

## LDL cholesterol metabolism

Of the 56 loci identified so far, a number affect lipid metabolism as an intermediary step, for example the LDL cholesterol receptor (*LDLR*) or *LPA* loci (Table 2). Interestingly, some of the genes found to associate with hypercholesterolaemia and CAD had never been implicated in these disorders before. Consequently, the novel loci suggest unexpected mechanisms affecting lipid metabolism. A prominent example is sortilin 1, encoded by the *SORT1* locus on chromosome 1 (Samani *et al*, 2007; Musunuru *et al*, 2010). Another example is *PCSK9*, which is discussed in more detail below because of the striking therapeutic options (Fig 3A).

## Triglyceride metabolism

**Figure 4:**
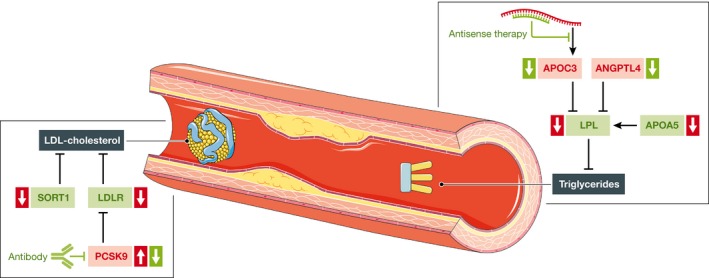
Novel insights into the genetic variation in LDL cholesterol metabolism and therapeutic modulation In low‐density lipoprotein (LDL) metabolism, sortilin 1 (SORT1), LDL cholesterol receptor (LDLR) and proprotein convertase subtilisin/kexin type 9 (PCSK9) are exemplarily shown (green, favourable effect regarding LDL cholesterol/triglycerides; red, unfavourable effect regarding LDL cholesterol/triglycerides; *↑*, variants increase the risk of CAD;* ↓*, variants decrease the risk of CAD).

Lipoprotein lipase (LPL), also genome‐wide significantly associated with CAD, seems to be a key player in CAD genetics, too. Under physiological conditions, LPL reduces triglyceride levels via the hydrolysis of lipoprotein‐bound triglycerides. LPL activity is increased by apolipoprotein A‐V (APOA5), but reduced by apolipoprotein C‐III (APOC3) and angiopoietin‐like 4 (ANGPTL4), all of which have been associated with CAD in a genome‐wide significant fashion (Table 3). It has been mentioned above that the *ANGPTL4* gene has just recently been identified in an exome‐wide association study to be associated with CAD. Indeed, the protective allele p.E40K represents a loss‐of‐function variant. Our consortium was able to demonstrate that this and other loss‐of‐function variants led to significantly reduced triglyceride levels, whereas LDL and HDL cholesterol levels were not affected. In accordance, loss‐of‐function variants were associated with a lower risk for CAD. This becomes even more apparent since we also detected a missense variant in the *LPL* gene, which led to a 20% reduction in LPL activity, to be associated with increased risk for CAD (Stitziel *et al*, 2016). The findings for *LPL* and *ANGPTL4* confirm recent results for two other regulators of LPL activity, APOA5 and APOC3, in that rare *APOA5* mutations increase both plasma triglyceride levels and risk of CAD (Do *et al*, 2015), whereas rare loss‐of‐function mutations in the *APOC3* gene have opposite effects (The TG and HDL Working Group of the Exome Sequencing Project, National Heart, Lung, and Blood Institute, 2014). Taken together, LPL activity seems to have a central role in triglyceride metabolism (Fig 3A) and consecutively CAD risk (Fig 4). Therefore, these proteins could represent possible targets for therapeutic intervention. Accordingly, attention should be paid to ongoing studies regarding *APOC3* inhibitors, which are discussed in more detail below.

## Blood pressure

Thanks to extensive genotyping efforts, several genomic loci have been identified to be associated with blood pressure. A score build on the cumulative effects of these loci displays a strong association with CAD (Levy *et al*, 2009; Newton‐Cheh *et al*, 2009; International Consortium for Blood Pressure Genome‐Wide Association Studies *et al*, 2011). Among the loci identified, there are several which are also genome‐wide significantly associated with CAD (Table 2, Fig 3B). It remains, however, unclear to which extent the effect on CAD is due to an increase in blood pressure given that the effect on blood pressure for each locus is very small (Lieb *et al*, 2013).

## NO/cGMP signalling

Nitric oxide (NO) is an important signalling molecule (Fig 3C) in the cardiovascular system, and the expression of endothelial NO synthase (eNOS), which produces NO from arginine, is reduced in atherosclerosis (Kawashima, 2004). As NO inhibits proatherogenic mechanisms such as platelet aggregation, smooth muscle cell proliferation/migration and adhesion of inflammatory cells (Davignon, 2004), it is plausible that a lack of NO itself is deleterious in the context of atherosclerosis. Furthermore, uncoupling of eNOS, for example when levels of the NO precursor arginine are reduced, may produce reactive oxygen species instead of NO, further promoting atherosclerosis (Kawashima, 2004). Interestingly, the *NOS3* gene has just recently been identified to be associated with CAD (Nikpay *et al*, 2015). Additionally, there is evidence that the particular variant (rs3918226), which is located in the promoter of the *NOS3* gene, influences the expression of eNOS with a negative effect of the risk allele (Salvi *et al*, 2013). As briefly discussed above, the *NOS3* gene has also been associated with blood pressure (Salvi *et al*, 2012, 2013). Bearing in mind that NO leads to vasodilatation, this is plausible. The effects on CAD risk are, however, larger than one would expect if just being mediated via increased blood pressure.

The major NO receptor, soluble guanylyl cyclase (sGC), also seems to play a role in the genetics of CAD. Indeed, the α_1_‐subunit (*GUCY1A3*) of sGC was shown by the meta‐analysis of the CARDIoGRAMplusC4D consortium to harbour a common variant associated with CAD (CARDIoGRAMplusC4D Consortium *et al*, 2013). However, even more evidence comes from the detection of a rare loss‐of‐function variant identified in an extended family with high prevalence of premature CAD and MI (Erdmann *et al*, 2013), in which exome sequencing identified a digenic mutation in *GUCY1A3* and *CCT7* to be responsible for the phenotype. Furthermore, mice lacking the α_1_‐subunit of the sGC have been shown to display accelerated thrombus formation (Erdmann *et al*, 2013). Platelets indeed play an important role not only in atherothrombosis but also in atherosclerosis (Von Hundelshausen & Weber, 2007). Studies investigating the effect of sGC on plaque formation in mouse models are eagerly awaited. A protective role of sGC is thereby strongly suggested by studies showing that cGMP formation is reduced in neointimal lesions (Melichar *et al*, 2004) and that cGMP leads to down‐regulation of the expression of adhesion molecules for the recruitment of leucocytes into atherosclerotic plaques (Ahluwalia *et al*, 2004). The common variant associated with CAD (rs7692387) is located in an intron however, and the exact mechanism from genotype to phenotype at this locus thus far remains elusive. Like *NOS3*, the *GUCY1A3* gene is also associated with blood pressure (International Consortium for Blood Pressure Genome‐Wide Association Studies *et al*, 2011).

## Vascular remodelling

Arterial remodelling is a crucial process in atherosclerosis (Fig 3D). Interestingly, constrictive remodelling and expansive remodelling of arteries exhibit different plaque morphologies: constrictive remodelling accompanied by lumen narrowing presents with more stable plaques, whereas expansive remodelling does not feature lumen narrowing but is associated with rather unstable plaques (Pasterkamp *et al*, 1998; Smits *et al*, 1999). A novel mechanism linking these observations might involve ADAMTS‐7, an extracellular matrix (ECM) protease. Variants in *ADAMTS7* have been identified to be associated with CAD (Coronary Artery Disease C4D Genetics Consortium, 2011; Reilly *et al*, 2011; Schunkert *et al*, 2011). *ADAMTS7* was more strongly associated with CAD rather than MI, suggesting a major role in plaque formation and not in plaque rupture (Reilly *et al*, 2011). At that time, little was known about *ADAMTS7* in cardiovascular diseases. One study had shown that the expression of the rat counterpart Adamts‐7 is found to be increased in carotid arteries secondary to balloon injury (Wang *et al*, 2009). This effect was supposed to lead to the degradation of an abundantly expressed ECM protein, cartilage oligomeric matrix protein (COMP). COMP itself had been shown to affect vascular smooth muscle cells with intact COMP maintaining a differentiated, contractile phenotype (Wang *et al*, 2010). In the meantime, two studies investigating the role of *ADAMTS7* in mouse models revealed evidence that mice lacking Adamts‐7 (*Adamts7* ^−/−^) display reduced atherosclerotic plaque formation compared to WT littermates when they are on a proatherogenic background and fed a western diet. This effect was most prominent in *Adamts7* ^−/−^ *Ldlr* ^−/−^ mice with a strong reduction in plaque formation in the aortic root as well as in the *en face* whole aorta analysis (Bauer *et al*, 2015). The mechanism underlying this phenotype is still unclear. The second study focused on vascular remodelling. *Adamts7* ^−/−^ mice displayed strongly reduced neointima formation after wire‐mediated vascular injury (Bauer *et al*, 2015; Kessler *et al*, 2015), a phenotype that is supposed to be mainly mediated by beneficial effects in endothelial cells (Kessler *et al*, 2015). Endothelial cells of *Adamts7* ^−/−^ mice showed increased proliferation and migration *in vitro*. In accordance, reendothelialization of injured arteries was significantly accelerated in *Adamts7* ^−/−^ compared to WT mice. This effect is thought to be due to the inhibition of the degradation of a novel ADAMTS‐7 target—thrombospondin‐1 (TSP‐1)—into bioactive fragments (Kessler *et al,* 2015). The concept of cell–matrix and ECM‐mediated cell–cell communication via finely regulated expression of proteases is attractive, especially regarding ECM proteins that contain several domains with differential functions such as TSP‐1 (Iruela‐Arispe *et al*, 1999). Interestingly, *TSP1* has also been investigated in candidate gene studies in CAD (Stenina *et al*, 2007). As for many other candidates that have been investigated in the pre‐GWAS era, for example the *ACE* gene (Mayer *et al*, 2007), no signal has been detected for the *TSP1* gene in GWAS so far.

## Inflammation

Inflammation is a central feature of atherosclerosis (Fig 3E) (Swirski & Nahrendorf, 2013). This also holds true for its genetic component. Indeed, the genes encoding the cytokine *CXCL12* and the interleukin 6 receptor (*IL6*) have been associated with CAD. In line, a pathway analysis of the CARDIoGRAMplusC4D meta‐analysis has also shown a major role for variants in inflammatory genes (CARDIoGRAMplusC4D Consortium, 2013). By contrast, C‐reactive protein (CRP) appears to be rather a marker than a causal factor, as its genetic variation does not translate to CAD risk (Schunkert & Samani, 2008; Zacho *et al*, 2008; Elliott *et al*, 2009; C Reactive Protein Coronary Heart Disease Genetics Collaboration (CCGC) (2011)).

Novel insights might come from functional investigation of the *SH2B3*/*LNK* gene. The variant rs3184504 tagging the *SH2B3* gene is associated with CAD (Gudbjartsson *et al*, 2009; CARDIoGRAMplusC4D Consortium, 2013). The interpretation of this association is complicated by the fact that this particular variant also represents an eSNP for a neighbour gene, *ATXN2* (Braenne *et al*, 2015). Nevertheless, the *SH2B3* gene, which encodes an adaptor protein expressed, for example, in leucocytes and platelets, revealed in a knock‐out mouse model (*Lnk* ^−/−^) increased platelet counts, but reduced thrombus stability (Takizawa *et al*, 2010). Bearing in mind that the risk allele at the *SH2B3* locus is also associated with increased platelet count (Soranzo *et al*, 2009), altered Sh2b3/Lnk function—as present in the mouse—seems to be a suitable model to analyse the functional impact of the variant in CAD. Besides the platelet phenotypes (Soranzo *et al*, 2009; Takizawa *et al*, 2010) and the also known association with blood pressure (Levy *et al*, 2009; Newton‐Cheh *et al*, 2009), *SH2B3* seems to mainly influence inflammatory processes. From a genetic point of view, this is already noticeable from the associations of the locus with different inflammatory diseases, for example coeliac disease (Hunt *et al*, 2008), type 1 diabetes (Barrett *et al*, 2009; Concannon *et al*, 2009) and high eosinophil numbers (Gudbjartsson *et al*, 2009). An experimental study revealed that Sh2b3/Lnk influences the activation of dendritic cells, thereby modulating the immune response, that is dendritic cells from *Lnk* ^−/−^ mice are hyper‐responsive to IL‐15, resulting in an excessive production of IFN‐γ (Mori *et al*, 2014). So far, the results regarding atherosclerotic phenotypes are still lacking. In a rat myocardial infarction model, however, knockout of *Sh2b3*/*Lnk* led to significantly increased fibrosis and leucocyte infiltration, resulting in reduced cardiac function (Flister *et al*, 2015). Taken together, genetic and experimental results point to a role for *SH2B3*/*LNK* in inflammatory processes in CAD, making SH2B3/LNK a promising candidate for therapeutic interventions.

## Therapeutic strategies

One major aim of the efforts to elucidate the genetic basis of CAD and MI is the identification of novel targets and the consequent development of novel strategies in prevention and therapy. Of interest, targets identified by genetic studies are estimated to be twice as successful in the development of new drugs compared to those identified by other means (Barrett *et al*, 2015; Nelson *et al*, 2015).

As discussed above, some genetic variants influence traditional risk factors, such as blood pressure and lipid metabolism, which may be targets for treatment themselves. In addition, based on genetic discoveries, targeted novel therapies may become available for focussed interference with lipid metabolism or currently unexploited mechanisms.

## Novel therapeutics in lipid metabolism

LPA, PCSK9, LPL, ANGPTL4, APOA5 and APOC3 are several promising targets in lipid metabolism available for therapeutic interference (Fig 4). PCSK9 is the most established one, since it has been known for more than a decade that gain‐of‐function and loss‐of‐function variants in the *PCSK9* gene increase (Abifadel *et al*, 2003) and decrease (Cohen *et al*, 2006) the risk of CAD and MI, respectively. GWAS also identified an association between common variants in the *PCSK9* gene and both cholesterol levels (Teslovich *et al*, 2010) and CAD (Myocardial Infarction Genetics Consortium *et al*, 2009). PCSK9 functions in LDL cholesterol metabolism by tagging for the degradation of intracellular as well as extracellular LDL cholesterol receptors (Brautbar & Ballantyne, 2011). Thus, current therapeutic concepts exploit monoclonal antibodies to inhibit the effect of PCSK9 in the circulation but also RNAi‐ and small molecule‐based approaches are in the development and evaluation. Table 4 provides an overview over the compounds currently under investigation using PCSK9 as target.

**Table 4:** Currently investigated PCSK9 inhibitors

Name	Mechanism of action	Phase of development	Approved	LDL
Evolocumab^a^	Human mAb	Phase IV	Yes^e^	↓ 61% (Sabatine *et al*, 2015)
Alirocumab^b^	Human mAb	Phase IV		↓ 62% (Robinson *et al*, 2015)
Bococizumab^c^	Humanized mAb (Liang *et al*, 2012)	Phase II	No	↓ 21–54% (Ballantyne *et al*, 2015)
ALN‐PCS^d^	Antisense oligo (Frank‐Kamenetsky *et al*, 2008)	Phase I	No	↓ 40% (Fitzgerald *et al*, 2014)
NA	VLP‐based vaccine (Crossey *et al*, 2015)	Preclinical	No	NA

mAb, monoclonal antibody; VLP, virus‐like particle; LDL., LDL cholesterol reduction; NA, not available.

a Amgen.

b Regeneron, Sanofi.

c Pfizer.

d Alnylam, The Medicines Company.

e Approved for familial hypercholesterolaemia, statin intolerance and insufficient LDL cholesterol control with statins.

LPL and the proteins regulating its activity are also attractive targets for novel treatment strategies. As discussed above, APOC3 reduces LPL activity. Accordingly, inhibition of APOC3 by RNAi‐mediated knockdown is currently being investigated in clinical trials. In a dose‐ranging phase II study, ISIS 304801, an antisense APOC3 inhibitor, led to a dose‐dependent 31–71% reduction in triglycerides (Gaudet *et al*, 2015). Also *ANGPTL4*, a gene recently identified by an exome‐wide association study and also involved in the regulation of LPL activity, may be the target of novel drug approaches: mice deficient for *Angptl4* display reduced triglyceride levels mainly due to increased clearance and reduced production of very low‐density lipoproteins (Desai *et al*, 2007).

## Targeting NO/cGMP signalling

The NO/cGMP pathway appears to play an important role in atherosclerosis since two key enzymes, that is eNOS and sGC, are genome‐wide significantly associated with CAD (CARDIoGRAMplusC4D Consortium, 2013; Nikpay *et al*, 2015). Additionally, the importance of the pathway has been confirmed by the fact that loss‐of‐function mutations affecting the function of the sGC are responsible for premature CAD and MI in an extended family (Erdmann *et al*, 2013). Whereas the mechanism involving the common variants remains to be elucidated, several therapeutic options are already available. Although increasing the availability of NO might not be achieved the expected success due to adverse effects and pharmacokinetic obstacles, targeting the sGC directly could solve these problems at least in part. Riociguat is a sGC stimulator already in clinical use. In the PATENT trial, the drug has proven efficacy in patients with pulmonary hypertension (Ghofrani *et al*, 2013). Riociguat is also very interesting because it acts synergistically with NO (Stasch & Hobbs, 2009); that is, reduced sGC activity or expression could hypothetically be compensated by the presence of such modulators of sGC activity. Future experimental studies are needed to explore the effect of sGC stimulators on atherosclerosis phenotypes.

## Precision medicine

One possibility of improving treatment is the identification and evaluation of novel drug targets. A second way to exploit GWAS findings is to develop individualized treatment strategies. The development of so‐called *precision medicine* is a major goal of current funding concepts (Jameson & Longo, 2015). Unfortunately, there is currently only limited evidence to suggest that genetic testing of individual common risk variants allows for stratification into different treatment modalities. However, individuals with a high genetic risk score appear to have a larger benefit from statin treatment than those with low genetic risk (Hughes *et al*, 2012; Mega *et al*, 2015; Schunkert & Samani, 2015). Further, stimulating input in this field might come from systems medicine approaches (Bjoerkegren *et al*, 2015). For example, an integrative analysis revealed an enrichment of CAD‐associated variants in differentially co‐expressed microRNA–mRNA pairs (Huan *et al*, 2013).

## Outlook

GWAS have led to the identification of more than 50 genetic variants associated with CAD and MI so far, and likely more will follow. These variants reflect an important progress in defining the mechanisms leading to disease. However, the variants identified thus far only account for no more than 10% of the heritable risk. Future studies will aim to detect rare variants with strong impact on disease risk and might thus close this gap. Additionally, the field of epistasis, that is the interaction of different genes, is rapidly evolving. Finally, it has been shown for other complex disorders that the combined small effects of hundreds of thousands of SNPs may jointly explain the currently poorly understood inheritance patterns (Yang *et al*, 2015). The results achieved thus far do, however, provide valuable insight into the pathophysiology of CAD and MI and are starting points for individualized treatment strategies.

## Conflict of interest

The authors declare that they have no conflict of interest.

**Pending issues**

- How can our understanding of the heritability of coronary artery disease be improved?
- To which extent do the interactions of specific loci contribute to the heritability of the disease?
- What are the molecular mechanisms by which non‐coding regions affect risk of coronary artery disease?
- How can the acquired knowledge be translated into improved prevention and therapeutic strategies?
